# Cell Membrane Disruption Stimulates NO/PKG Signaling and Potentiates Cell Membrane Repair in Neighboring Cells

**DOI:** 10.1371/journal.pone.0042885

**Published:** 2012-08-07

**Authors:** Tatsuru Togo

**Affiliations:** Department of Anatomy, St. Marianna University School of Medicine, Kawasaki, Japan; Medical College of Georgia, United States of America

## Abstract

Resealing of a disrupted plasma membrane at the micron-diameter range requires Ca^2+^-regulated exocytosis. Repeated membrane disruptions reseal more quickly than the initial wound, and this potentiation of membrane resealing persists for at least 24 hours after the initial wound. Long-term potentiation of membrane resealing requires CREB-dependent gene expression, which is activated by the PKC- and p38 MAPK-dependent pathway in a wounded cell. The present study demonstrates that membrane resealing is potentiated in both wounded and neighboring cells in MDCK cells. Wounding of cells expressing CREB133, a mutant variant of CREB, does not show the potentiated response of cell membrane resealing in either wounded or neighboring cells. Furthermore, wounding of cells induces CREB phosphorylation, not only in wounded cells, but also in neighboring cells. Inhibition of the nitric oxide/PKG signaling pathway suppresses CREB phosphorylation in neighboring cells, but not in wounded cells. The potentiation of membrane resealing in neighboring cells is suppressed if the nitric oxide/PKG pathway is inhibited during the initial wound. Together, these results suggest that the nitric oxide/PKG pathway stimulates CREB phosphorylation in neighboring cells so that subsequent cell membrane disruptions of the neighboring cells reseal more quickly.

## Introduction

Mechanical stress induces disruptions of the plasma membranes of cells in various animal tissues under physiological conditions, and cells survive these disruptions by resealing the cell membrane [1]. Mechanisms for membrane resealing may differ depending on the size of the lesion. For large cell membrane lesions, homotypic vesicle-vesicle fusion may occur to create a membrane “patch” [2], [3]. However, small micron-diameter disruptions evoke the Ca^2+^-dependent exocytosis of vesicles near the wound site, which is essential for successful membrane resealing [4], [5], [6], [7], [8], [9], [10]. This small disruption reaction has a lower Ca^2+^ threshold than that for “patch” formation [1]. It has been proposed that wound-induced exocytosis promotes resealing by lowering the plasma membrane tension [11]. In addition to mechanical disruption of the plasma membrane, cells also respond to the other form of lesions. For example, stable lesions induced by bacterial pore-forming toxin are removed from the cell membrane by endocytosis [12].

Previously, it was demonstrated that exocytosis induced by small micron-diameter disruptions is potentiated following an initial wound, and repeated membrane disruptions reseal more quickly than the initial wound [8], [13]. This response is protein kinase C and protein kinase A-dependent in the early stages (minutes), in the intermediate term (hours) requires protein synthesis, and for long term (24 hours) depends on the activation of a transcription factor, cAMP response element-binding protein (CREB) [8], [13], [14]. Furthermore, the activation of CREB in a wounded cell requires a PKC- and p38 MAPK-dependent signaling pathway [15].

In multicellular organisms, cells communicate with each other through hundreds of signaling molecules including proteins, small peptides, nucleotides, steroids, and gas, such as nitric oxide (NO). Disruptions of plasma membranes are widespread, common, and normal events in many animal tissues, and these disruptions stimulate various cellular responses [1]. Thus, cell membrane disruption may induce intercellular signaling, in addition to intracellular signaling. In fact, mechanically scratching cell monolayers induces intercellular Ca^2+^ waves [16], and dysferlin mediates Ca^2+^-triggered intercellular signaling in response to membrane disruption [17]. Although cell-cell signaling has been extensively studied, it is unclear how neighboring cells respond to these signals. The aim of the present study was to investigate whether CREB-dependent long-term potentiation of membrane resealing propagates to neighboring cells via a cell-cell signaling pathways. The results revealed a signaling pathway that leads to CREB phosphorylation and potentiation of membrane resealing in neighboring cells.

## Results

### Cell Membrane Disruption Potentiates Cell Membrane Resealing in both Wounded and Neighboring Cells in a CREB-dependent Manner

**Figure 1:**
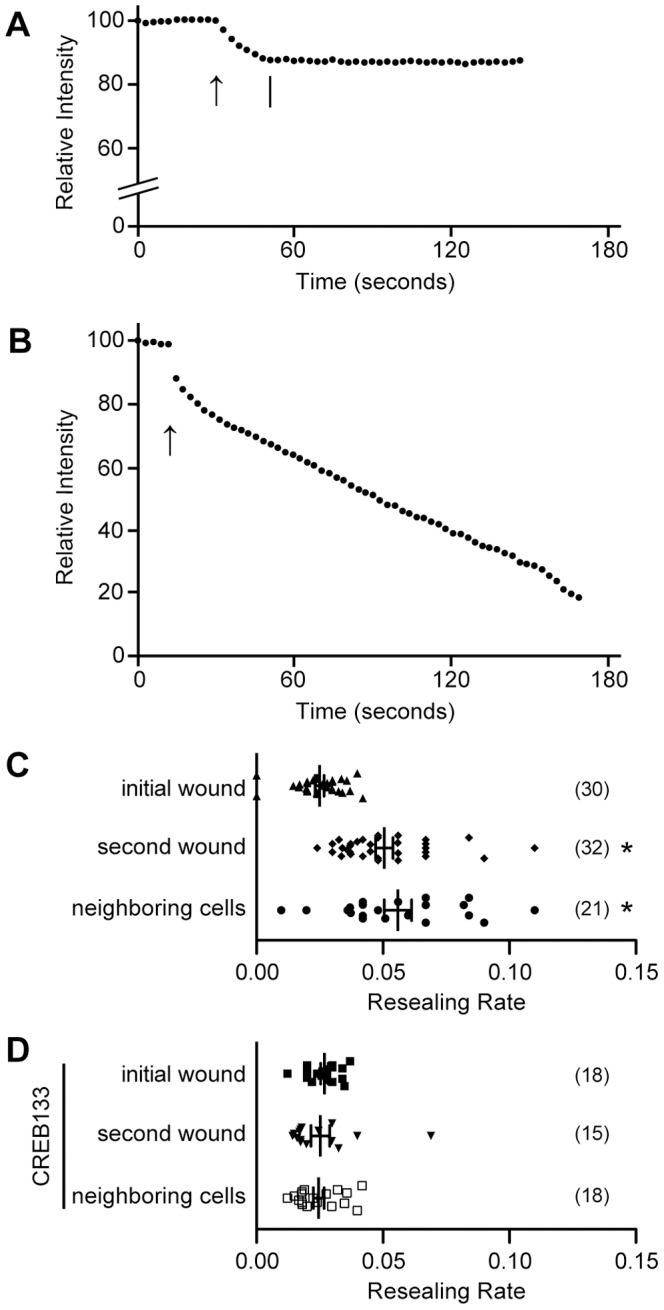
Cell membrane disruption potentiates membrane resealing in both wounded and neighboring cells in a CREB-dependent manner. (A) A typical recording of the resealing assay. To mark the wounded cells, cells were scratched in the presence of Alexa 488-dextran in 1.8 mM Ca^2+^ Ringer's solution, and returned to normal culture conditions. Cells were loaded with calcein red-orange AM 24 hours after the scratching, and the changes in fluorescent intensity of calcein red-orange upon cell membrane disruption by a glass needle were monitored in 1.8 mM Ca^2+^ Ringer's solution. Units of fluorescence intensity were normalized to 100% before wounding. Arrow indicates the time of membrane disruption by a glass needle. Bar indicates the completion time of membrane resealing. (B) When changes in fluorescent intensity were monitored in 0.1 mM Ca^2+^ Ringer's solution that inhibits membrane resealing, the intensity was persistently decreased. (C) Comparison of membrane resealing rates of wounded and neighboring cells. The resealing rate was defined as the reciprocal of the resealing time in seconds. For cells that failed to reseal, the rate was defined as zero. Bars are mean ± s.e.m. Each point represents one measurement. Numbers of cells observed are indicated in parentheses. *, P<0.01 versus initial wound. (D) Comparison of the resealing rates of wounded and neighboring cells expressing CREB133. Bars are mean ± s.e.m. Each point represents one measurement. Numbers of cells observed are indicated in parentheses.

To investigate whether membrane resealing is potentiated in both wounded and neighboring cells, MDCK cells were initially wounded by scratching in 1.8 mM Ca^2+^ Ringer's solution containing Alexa 488-dextran. Alexa 488-dextran enters cells that incur cell membrane disruption, and is retained in wounded cells that successfully reseal. Membrane resealing was analyzed 24 hours later as described in the Materials and Methods. Briefly, cells were loaded with 1 µM calcein red-orange AM 24 hours after scratching. Then cells were wounded using a glass needle in 1.8 mM Ca^2+^ Ringer's solution, and the changes in fluorescent intensity of calcein red-orange were monitored. In this assay, the cells adjacent to the wounded cells were identified as neighboring cells. As described previously [18], cell membrane disruption (arrows in Figure 1A and B) is indicated by a decrease in the fluorescence intensity of the dye. In most cases, the decrease in fluorescence intensity stopped (bar in Figure 1A). On the other hand, when the cells were wounded under the condition that inhibits membrane resealing (low-Ca^2+^) as a control, the intensity was persistently decreased (Figure 1B), indicating that successful membrane resealing results in transient decrease in fluorescent intensity as reported previously [18]. To compare the timing of membrane resealing in each condition, the resealing time was measured. Since it was impossible to determine resealing time in the cells that failed to reseal as shown in Figure 1B, reciprocal of resealing time was calculated. These values were termed “resealing rates” as reported previously, and the value was defined as zero for cells that failed to reseal [4], [8], [11], [13], [18], [19].

When non-scratched cells were initially wounded in 1.8 mM Ca^2+^ Ringer's solution, the resealing rate was 0.025±0.002 (n = 30) (Figure 1C), and relative fluorescent intensity of calcein red-orange was decreased to 79.1±0.83% (n = 30) when cell membrane resealed. On the other hand, the resealing rate for previously wounded cells increased significantly to 0.051±0.003 (n = 32) (Figure 1C). Furthermore, fluorescent intensity of calcein was 89.2±0.65% (n = 32) at the time of membrane resealing, indicating that faster membrane resealing resulted in lesser loss of cellular constituents. These results are consistent with that of a previous report that showed existence of long-term potentiation of membrane resealing in 3T3 fibroblasts [13]. Furthermore, the resealing rate of neighboring cells also increased significantly to 0.056±0.005 (n = 21) (Figure 1C), and the intensity of calcein red-orange was 87.6±1.6% (n = 21) when cell membrane resealed. These results indicate that membrane resealing of neighboring MDCK cells is potentiated by initial wounding, as are directly wounded MDCK cells.

Long-term potentiation of membrane resealing in a wounded cell has been shown to require the transcription factor CREB in 3T3 fibroblasts [13], [15]. To investigate whether long-term potentiation of membrane resealing requires CREB in neighboring cells, MDCK cells were transfected with a dominant negative CREB construct, pCMV-CREB133, and a stable transfectant was selected at 0.4 mg/ml G418. Cells transfected with pCMV-CREB133 constitutively expressed a mutant variant of CREB that contains a serine to alanine mutation corresponding to amino acid 133. When non-scratched cells were loaded with calcein red-orange AM, and were initially wounded in 1.8 mM Ca^2+^ Ringer's solution, the resealing rate was 0.027±0.002 (n = 18) (Figure 1D), and the relative fluorescent intensity of calcein was decreased to 78.9±1.1% (n = 18) when cell membrane resealed. These values showed no significant differences from the values for initial wounding of non-transfected cells, indicating that the expression of CREB133 had no deleterious effect on membrane resealing, as reported previously [13]. In the separate dishes, cells were wounded by scratching in 1.8 mM Ca^2+^ Ringer's solution in the presence of the marker dye Alexa 488-dextran. These cells were loaded with calcein red-orange AM 24 hours after scratching, and the rates of membrane resealing and intensity of calcein red-orange at the time of membrane resealing were analyzed. As shown in Figure 1D, membrane resealing was not potentiated in either wounded or neighboring cells. The resealing rates for previously wounded and neighboring cells were 0.025±0.004 (n = 15) and 0.025±0.002 (n = 18), respectively. Furthermore, the fluorescent intensities of calcein red-orange at the time of cell membrane resealing were 79.4±0.94% (n = 15) and 80.2±1.0% (n = 18), respectively, and these values had no significant differences from the value for initial wounding of CREB133-expressing cells. These results indicate that the potentiated response of membrane resealing in wounded cells as well as the neighboring cells requires CREB in MDCK cells.

### Cell Membrane Disruption Induces Phosphorylation of CREB in both Wounded and Neighboring Cells

**Figure 2:**
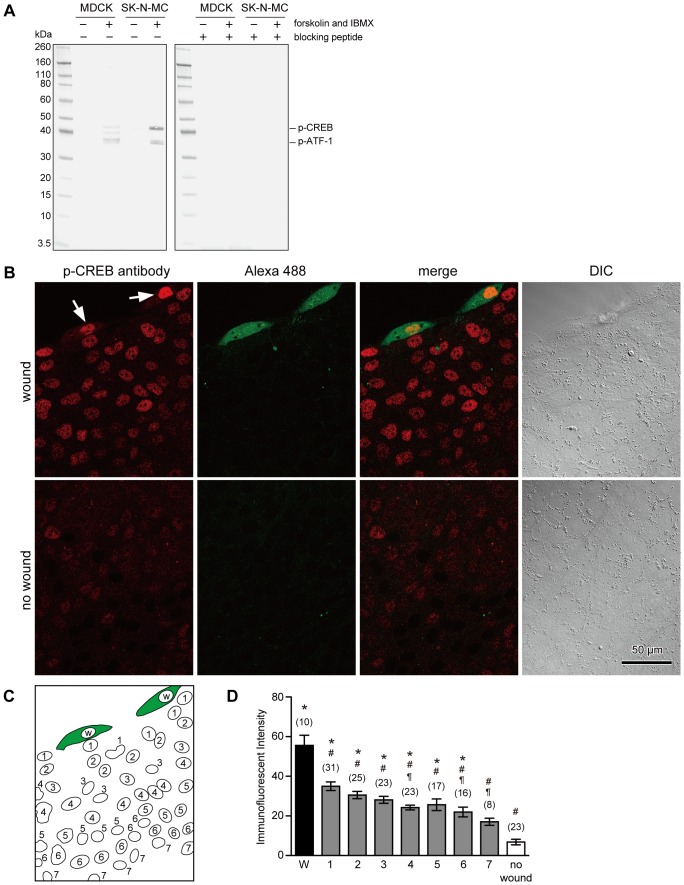
Cell membrane disruption induces phosphorylation of CREB, not only in wounded cells, but also in neighboring cells. (A) Western blot analysis of extracts from MDCK and SK-N-MC cells, untreated or forskolin- and IBMX-treated, using anti-phospho-CREB (Ser133) (87G3) rabbit mAb with or without preincubation with phospho-peptide. Experiments were repeated three times. (B) Cells were wounded by scratching in the presence of fixable Alexa 488-dextran in 1.8 mM Ca^2+^ Ringer's solution, fixed 1 hour later, and then immunostained with anti-phospho-CREB antibody. Cells that were wounded and survived plasma membrane disruption show cytosolic labeling with the marker dye, Alexa 488-dextran. Immunostaining of the nuclear region with an anti-phospho-CREB antibody was observed in wounded cells (arrows). In addition to these cells, nuclear staining with anti-phospho-CREB antibody was also observed in neighboring cells. (C) Schematic drawing of nuclear localization in the image shown in (B). The nuclei of wounded cells were labeled as W, nuclei adjacent to Nucleus W were labeled as 1, nuclei adjacent to Nucleus 1 were labeled as 2, and so on. (D) Quantitative analysis of the immunostaining intensity of cell nuclei. Data were collected from three different images including (B), and are expressed as mean ± s.e.m. Numbers of cells observed are indicated in parentheses. *, P<0.01 versus “no wound”; #, P<0.01 versus W; ¶, P<0.01 versus Nucleus 1.

To observe the phosphorylation of CREB, anti-phospho-CREB (Ser133) (87G3) mAb was used in this study. Western blot analysis indicated that this antibody detects phosphorylated CREB and phosphorylated form of the CREB-related protein, ATF-1 in SK-N-MC cells as manufacturer described (Figure 2A). When the extracts were obtained from untreated or forskolin- and IBMX-treated MDCK cells, this antibody recognized phospho-CREB (43 kDa) and two other proteins including phospho-ATF-1 in stimulated cells (Figure 2A). If the antibody was preincubated with the phospho-peptide corresponding to residues surrounding Ser133 of CREB, reactivity of the antibody was completely blocked.

To observe the phosphorylation of CREB and CREB-related proteins in response to cell membrane disruption in MDCK cells, cells were scratched in the presence of the marker dye, fixable Alexa 488-dextran, in 1.8 mM Ca^2+^ Ringer's solution, fixed at 1 hour later, and then immunostained with anti-phospho-CREB antibody. Staining of the nuclear region by anti-phospho-CREB antibody was observed in cells containing Alexa 488-dextran (arrows in Figure 2B), suggesting that CREB and its related proteins are phosphorylated in wounded cells as reported previously in 3T3 fibroblasts [13], [15]. In addition to wounded cell, nuclear staining with anti-phospho-CREB antibody was also observed in neighboring cells (Figure 2B). To compare the degree of immunofluorescent intensities between wounded cells and neighboring cells, nuclei were numbered as in Figure 2C, and the average fluorescent intensities were measured. The data were collected from three different images that were obtained from three different woundings on different days. Wounding by scratching resulted in a significant increase in the immunostaining intensity of anti-phospho-CREB antibody in wounded cells and in cells that are up to six-cell distant from the wounded cell (Figure 2D). The greatest intensity was observed in the wounded cells, and the intensity of cells adjacent to wounded cell was 63% of that of the wounded cells. Among the neighboring cells, the immunofluorescent intensity gradually decreased with increased distance from wounded cells, and the intensity of Nucleus 1 was significantly higher than those of Nuclei 4, 6 and 7. These results suggest that intercellular signaling is stimulated upon cell membrane disruption to induce the phosphorylation of CREB and CREB-related proteins in neighboring cells that had not been directly injured.

### Inhibition of NO/PKG Signaling Pathway Suppresses Phospho-CREB Immunostaining in Neighboring Cells

**Figure 3:**
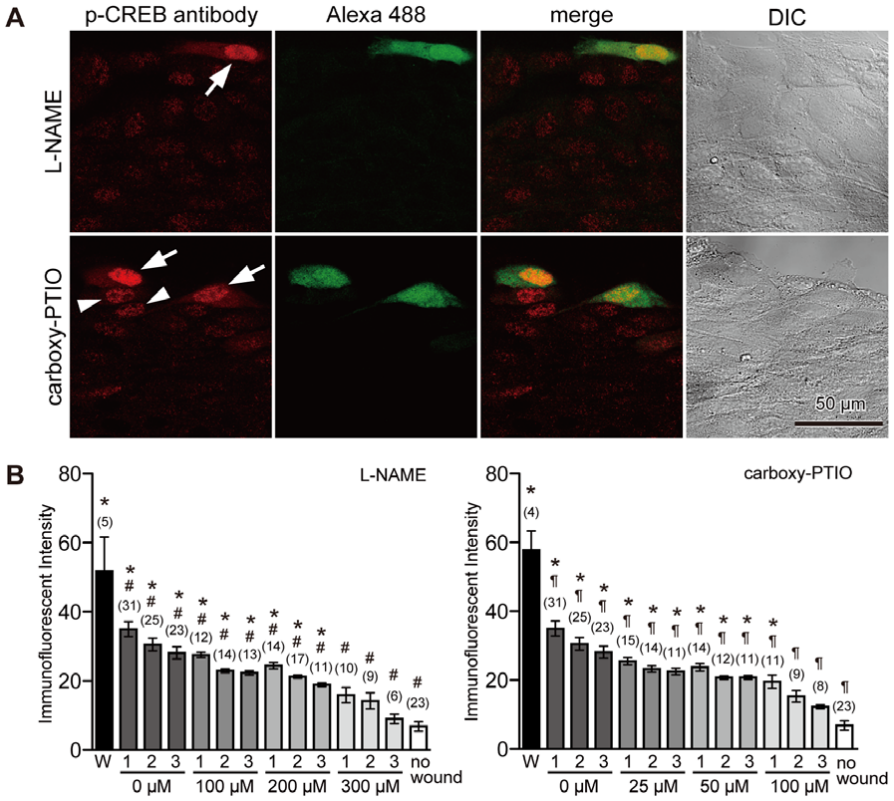
Inhibition of the NO signaling suppresses phospho-CREB immunostaining in neighboring cells, but not in wounded cells. (A) Cells were wounded in the presence of fixable Alexa 488-dextran along with 300 µM L-NAME or 100 µM carboxy-PTIO in 1.8 mM Ca^2+^ Ringer's solution, fixed 1 hour later, and then immunostained with anti-phospho-CREB antibody. Phosphorylation of CREB and CREB-related proteins was suppressed in neighboring cells, and only a few cells show weak staining with anti-phospho-CREB antibody (arrowheads), whereas phosphorylation of CREB and CREB-related proteins was induced in wounded cells (arrows). (B) Quantitative analysis of the immunostaining intensity of cell nuclei. Nuclei were numbered as Figure 2C, and the immunofluorescent intensities were measured. Data were collected from three different images at each treatment, and are expressed as mean ± s.e.m. Data for non-treated cells are from Figure 2D for comparison. Numbers of cells observed are indicated in parentheses. *, P<0.01 versus “no wound”; #, P<0.01 versus W (L-NAME); ¶, P<0.01 versus W (carboxy-PTIO).

Wounding may induce the production and/or release of diffusible molecules into the extracellular milieu to potentiate membrane resealing of neighboring cells. The present study investigated the involvement of NO as a candidate for intercellular signaling molecule. Cells were scratched in the presence of reagents that suppress NO signaling together with fixable Alexa 488-dextran in 1.8 mM Ca^2+^ Ringer's solution, fixed 1 hour later, and then immunostained with anti-phospho-CREB antibody. To block NO signaling, L-N^G^-nitroarginine methyl ester (L-NAME) and 2-(4-carboxyphenyl)-4,4,5,5-tetramethylimidazoline-1-oxyl-3-oxide (carboxy-PTIO) were used. L-NAME is commonly used to inhibit NO synthase, and carboxy-PTIO is a scavenger of NO [20]. When cells were initially wounded in the presence of either L-NAME (300 µM) or carboxy-PTIO (100 µM), phosphorylation of CREB and CREB-related proteins was induced in wounded cells (Figure 3A, arrows). Immunoreactive intensities of anti-phospho-CREB antibody in wounded cells were 51.8±9.9 (n = 5) and 57.8±5.5 (n = 4) for cells treated with L-NAME and carboxy-PTIO, respectively (Figure 3B), and there were no significant differences with that of controls (Figure 2D). However, in neighboring cells, the immunoreactivity of anti-phospho-CREB antibody was suppressed in a dose-dependent manner (Figure 3B). These results suggest that cell wounding stimulates NO signaling, which leads to phosphorylation of CREB and CREB-related proteins in neighboring cells.

**Figure 4:**
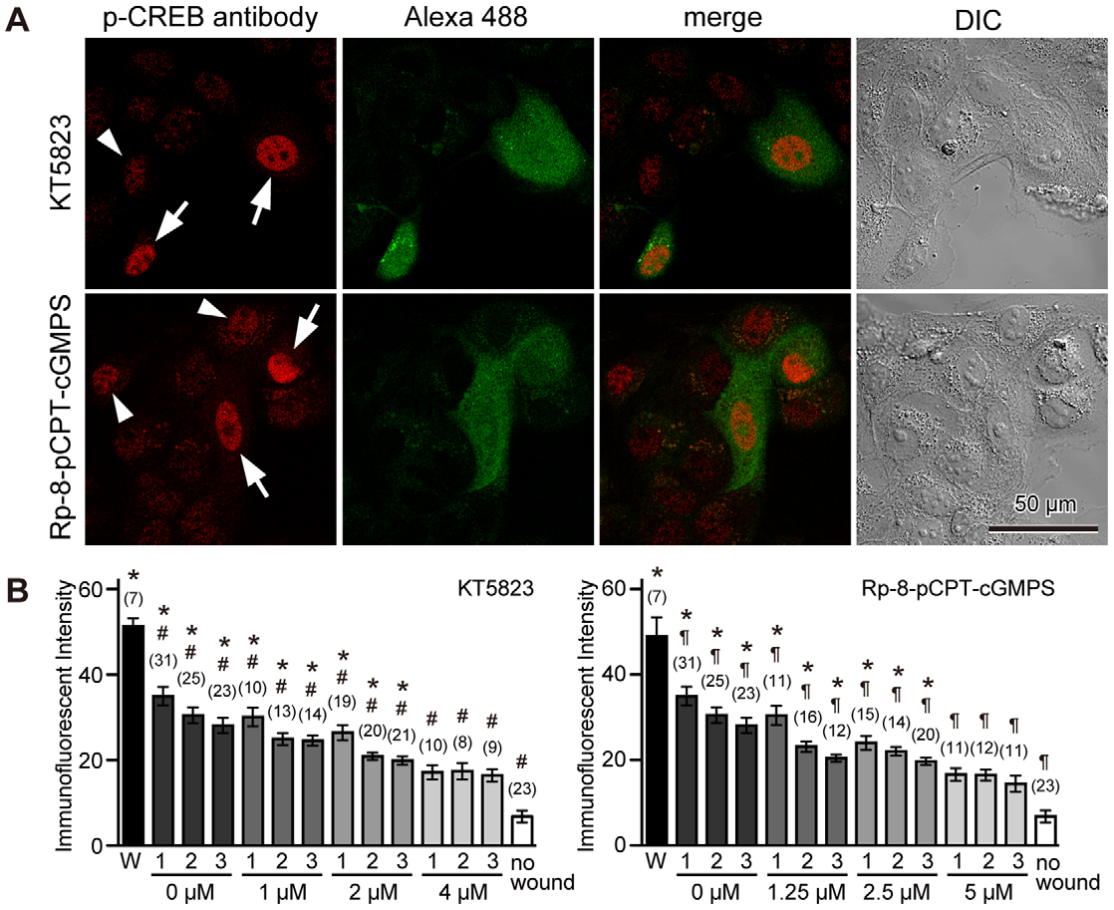
PKG inhibitors suppress phospho-CREB immunostaining in neighboring cells, but not in wounded cells. (A) Cells were wounded in the presence of fixable Alexa 488-dextran together with a PKG inhibitor, 4 µM KT5823 or 5 µM Rp-8-pCPT-cGMPS, in 1.8 mM Ca^2+^ Ringer's solution, fixed 1 hour later, and then immunostained with anti-phospho-CREB antibody. Phosphorylation of CREB and CREB-related proteins was induced in wounded cells (arrows). CREB phosphorylation was also observed in neighboring cells, but staining with anti-phospho-CREB antibody was weak (arrowheads). (B) Quantitative analysis of phospho-CREB immunostaining intensity of cell nuclei. Nuclei were numbered as in Figure 1B, and the immunofluorescent intensities were measured. Data were collected from three different images at each treatment, and are expressed as mean ± s.e.m. Data for non-treated cells are from Figure 2D for comparison. Numbers of cells observed are indicated in parentheses. *, P<0.01 versus “no wound”; #, P<0.01 versus W (KT5823); ¶, P<0.01 versus W (Rp-8-pCPT-cGMPS).

Many of the biological effects of NO are mediated by the activation of cGMP-dependent protein kinase (PKG) [21], [22]. To observe the effect of PKG inhibition on phosphorylation of CREB and CREB-related proteins upon cell membrane disruption, cells were scratched in the presence of two different PKG inhibitors, KT5823 or Rp-8-(4-Chlorophenylthio)-guanosine-3′,5′-cyclic monophosphorothioate (Rp-8-pCPT-cGMPS), together with fixable Alexa 488-dextran in 1.8 mM Ca^2+^ Ringer's solution. KT5823 is an ATP-competitive inhibitor [23], and Rp-8-pCPT-cGMPS antagonizes PKG activation by cGMP [24]. The scratched cells were fixed 1 hour later, and immunostained with anti-phospho-CREB antibody (Figure 4A). When cells were initially wounded in the presence of PKG inhibitors, phosphorylation of CREB and CREB-related proteins was induced in wounded cells (Figure. 4A, arrows). Immunoreactive intensities of wounded cells were 52.7±2.3 (n = 5) and 48.9±4.5 (n = 7) for cells treated with KT5823 (4 µM) and Rp-8-pCPT-cGMPS (5 µM), respectively (Figure 4B), and there were no significant differences with that of the controls (Figure 2D). On the other hand, in neighboring cells, inhibitors induced a significant decrease in the immunostaining intensity of anti-phospho-CREB antibody in a dose-dependent manner (Figure 4B). These results suggest that PKG is involved in phosphorylation of CREB and CREB-related proteins specifically in neighboring cells.

**Figure 5:**
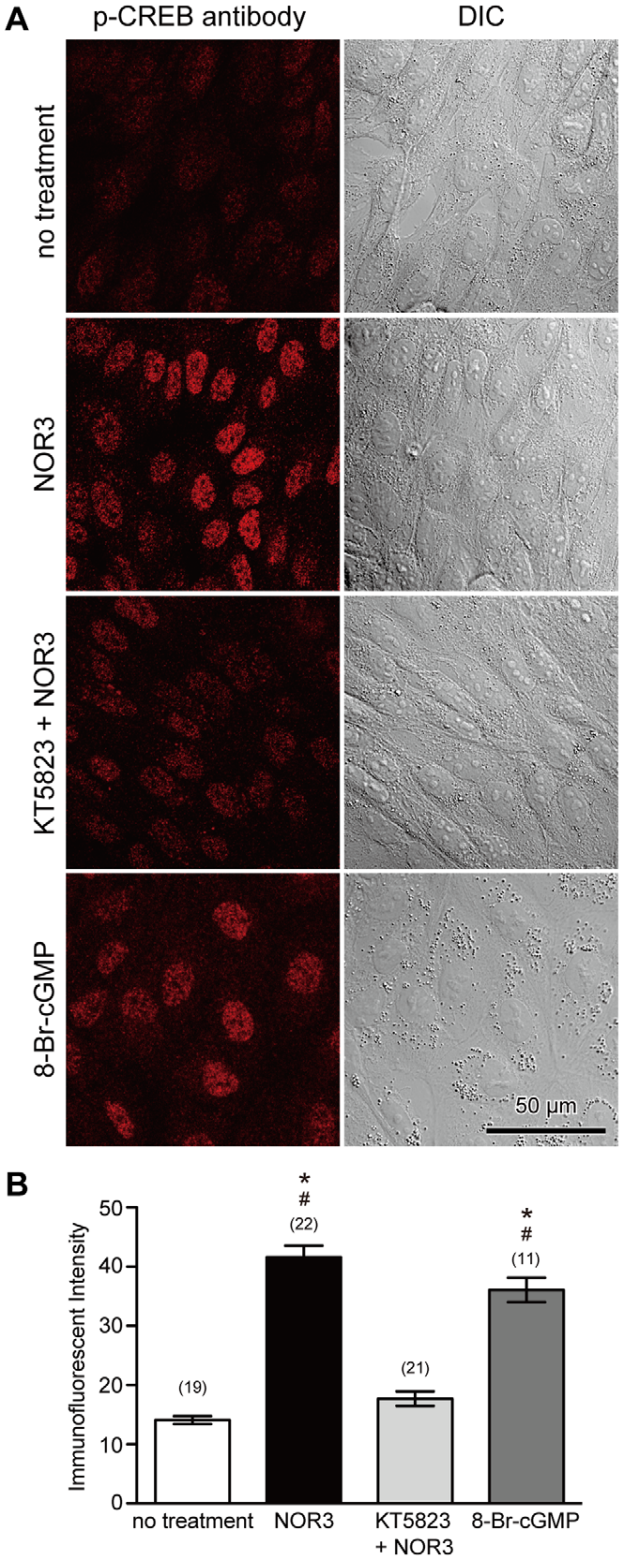
NO induces phosphorylation of CREB in a PKG-dependent manner. (A) Cells were treated with NOR3 (100 µM), a donor of NO, for 10 minutes in the absence or presence of a PKG inhibitor, KT5823 (4 µM), or treated with a cGMP analog, 8-Br-cGMP (5 µM), fixed 1 hour later, and then immunostained with anti-phospho-CREB antibody. Phosphorylation of CREB and CREB-related proteins was induced by NOR3 as indicated by immunostaining of nuclear regions with an anti-phospho-CREB antibody. However, KT5823 suppressed the effect of NOR3. Furthermore, 8-Br-cGMP induced CREB phosphorylation. (B) Quantitative analysis of immunostaining intensity of cell nuclei. Values are mean ± s.e.m. Numbers of cells observed are indicated in parentheses. *, P<0.01 versus no treatment; #, P<0.01 versus KT+NOR.

To confirm the involvement of the NO/PKG pathway in phosphorylation of CREB and CREB-related proteins, NO was applied to non-wounded cells using an NO-donating reagent, (±)-(*E*)-4-ethyl-2-[(*E*)-hydroxyimino]-5-nitro-3-hexenamide (NOR3), that releases 1.5–2 mole of NO per mole [25]. Cells were treated with 100 µM NOR3 for 10 minutes in the absence or presence of 4 µM KT5823, were fixed 1 hour later, and immunostained with anti-phospho-CREB antibody. As shown in Figure 5, the addition of extracellular NOR3 increased the intensity of phospho-CREB immunostaining. However, if cells were treated with NOR3 in the presence of 4 µM KT5823, immunostaining with anti-phospho-CREB antibody was suppressed (Figure 5). The application of 5 µM 8-bromoguanosine-cGMP (8-Br-cGMP), a membrane-permeable cGMP analog, also induced immunostaining with anti-phospho-CREB antibody (Figure 5). These results suggest that NO induces phosphorylation of CREB and CREB-related proteins via a PKG.

### Inhibition of NO/PKG Signaling Pathway Suppresses Potentiation of Membrane Resealing in Neighboring Cells

**Figure 6:**
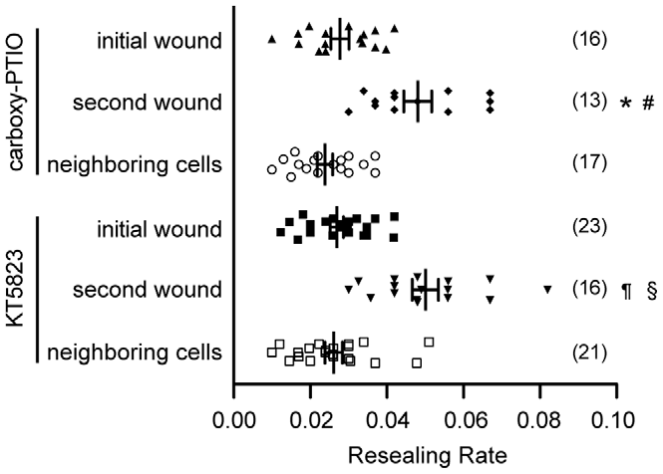
Inhibition of NO/PKG pathway suppresses potentiation of membrane resealing in neighboring cells. Cells were scratched in the presence of Alexa 488-dextran along with 100 µM carboxy-PTIO or 4 µM KT5823 in 1.8 mM Ca^2+^ Ringer's solution, and returned to normal culture conditions. These cells were loaded with calcein red-orange AM 24 hours after scratching, and the resealing rates were analyzed in 1.8 mM Ca^2+^ Ringer's solution. Bars are mean ± s.e.m. Each point represents one measurement. Numbers of cells observed are indicated in parentheses. Among the cells treated with carboxy-PTIO: *, P<0.01 versus initial wound; #, P<0.01 versus neighboring cells. Among the cells treated with KT5823: ¶, P<0.01 versus initial wound; §, P<0.01 versus neighboring cells.

To investigate whether the NO/PKG signaling is involved in long-term potentiation of membrane resealing in neighboring cells, cells were initially wounded by scratching in the presence of 100 µM carboxy-PTIO or 4 µM KT5823 in 1.8 mM Ca^2+^ Ringer's solution and marked with the dye Alexa 488-dextran. Then, the membrane resealing of wounded and neighboring cells was examined 24 hours later (Figure 6). When non-scratched cells were initially wounded in the presence of carboxy-PTIO and KT5823, the resealing rates were 0.028±0.009 (n = 16) and 0.027±0.008 (n = 23), respectively. Furthermore, the fluorescent intensities of calcein at the time of membrane resealing were 79.3±0.65% (n = 16) and 77.1±0.64% (n = 23), respectively. These values show no significant differences with the values observed in non-treated cells, indicating that both reagents had no deleterious effect on membrane resealing. When cells were initially wounded in the presence of carboxy-PTIO or KT5823, and wounded again 24 hours later, the resealing rates for a second wounding were increased significantly to 0.048±0.004 (n = 13) and 0.05±0.003 (n = 16), respectively. Furthermore, loss of calcein upon cell membrane disruption was suppressed, and the intensities at the time of membrane resealing were 87.5±0.88% (n = 13) and 91.4±0.78% (n = 16), respectively. However, in neighboring cells, the rate was suppressed to 0.024±0.002 (n = 17) and 0.026±0.002 (n = 21), respectively. Furthermore, the fluorescent intensities of calcein at the time of membrane resealing were 79.2±1.1% (n = 17) and 80.5±1.0% (n = 21), respectively. These results suggest that both NO and PKG are involved in long-term potentiation of membrane resealing in neighboring cells, but not in wounded cells.

## Discussion

Plasma membrane disruption at the micron-diameter range evokes a rapid exocytosis, which is essential for successful membrane resealing [1]. It has been previously demonstrated that repeated membrane disruption reveals long-term potentiation of Ca^2+^-regulated exocytosis in 3T3 fibroblasts, which is closely correlated with faster membrane resealing rates at repeated wounds [13]. This potentiation of exocytosis and membrane resealing depends on gene expression that is mediated by the transcription factor CREB via a PKC- and p38 MAPK-dependent pathway in a wounded cell. In addition to wounded cells, the present study demonstrates that wounding of MDCK cells potentiates membrane resealing in neighboring cells in a CREB-dependent manner. Since many organs in animals normally generates and/or receive considerable levels of mechanical stress repetitively, and since these stresses often result in the cell membrane disruptions [1], the multicellular adaptive response leading to faster cell membrane resealing at subsequent woundings may minimize the toxic effects of excessive Ca^2+^ influx into cells [26] and the loss of crucial cellular constituents from cells, and may protect tissues from mechanical stresses efficiently.

The antibody used in this study recognizes phosphorylated form of CREB-related proteins such as phospho-ATF-1 in addition to phospho-CREB. Thus immunofluorescent staining suggests that membrane disruption also induces phosphorylation of CREB-related proteins in addition to CREB. Although present study indicates that CREB is involved in the potentiated response of membrane resealing in both wounded and neighboring cells, it is not clear if CREB-related proteins such as ATF-1 are also involved in the potentiation of membrane resealing.

Previous studies did not detect CREB phosphorylation in neighboring cells in 3T3 fibroblasts [13], [15]. This may be due to wounding at low cell confluency and the analyses of CREB phosphorylation focused on wounded cells only. This inconsistency may also be due to the different type of cells used between those experiments and the present study, fibroblasts and epithelial cells. Thus, the responses to cell membrane disruption may vary depending on the cell type. It remains to be determined whether neighboring cells also respond to cell membrane disruption in fibroblasts.

In addition to CREB, cell membrane disruption also induces c-fos expression and nuclear translocation of NF-κB [27]. However, these two phenomena are reportedly restricted in the cell that suffered and resealed a cell membrane disruption [27]. Thus NO/PKG pathway stimulates CREB phosphorylation, but does not affect c-fos expression and nuclear translocation of NF-κB in neighboring cells.

The results of the present study strongly suggest that wound-induced exocytosis is potentiated in neighboring cells, since previous studies demonstrates that the amount of exocytosis evoked at a wound is closely correlated with the rate of membrane resealing in all cell types tested [4], [5], [6], [7], [8], [13]. In a preliminary study using MDCK cells, to observe exocytosis accompanying microdisruptions, the lipophilic fluorescent dye FM1-43 was preloaded by endocytosis overnight. FM1-43 intercalates into the outer leaflet of the lipid bilayers and is much more fluorescent in hydrophobic than in hydrophilic environments [28]. When cells are incubated with FM1-43, dye remaining in the plasma membrane rapidly diffuses away, leaving only dye that is trapped in the endocytosed membrane. Subsequent delivery of the FM1-43 into the plasma membrane by exocytosis allows diffusion of the dye into the external medium and results in a loss of cellular fluorescence. However, it was very difficult to detect FM1-43 destaining, especially in initial woundings in MDCK cells. One reason for this observation may be that the rate of membrane resealing for initial wounding is much slower than the other type of cells. When non-scratched MDCK cells were initially wounded, the resealing rate was 0.025±0.002 (n = 30) (Figure 1C). This value was significantly less than that of 3T3 fibroblasts (0.068±0.005, n = 55) [13] and corneal epithelial cells (0.065±0.002, n = 164) [14], suggesting that the amount of exocytosis upon cell membrane disruption in MDCK cells is much less than the other cells. Another possibility is that MDCK cells use vesicular compartments to reseal the cell membrane that are not labeled with FM1-43. Further studies are required to identify the vesicular compartment used in cell membrane repair before the amount of exocytosis is assessed.

It has been clearly demonstrated that wounding stimulates intercellular Ca^2+^ signaling [16], [17]. However it is not clear the relationship between NO/PKG signaling reported in this study and Ca^2+^ signaling. In addition to long-term, cells have a short-term response against cell membrane disruption [8], [13], [14]. Although this remains to be demonstrated, if neighboring cells have short-term responses upon cell membrane disruption, cells may use intercellular signaling such as NO/PKG and Ca^2+^ signaling. These will be the focus of further studies.

## Materials and Methods

### Cell Culture

MDCK cells were obtained from Health Science Research Resources Bank of Japan Health Sciences Foundation, and cultured in Dulbecco's modified Eagle's medium (Life Technologies) containing 10% fetal bovine serum (FBS) (Biowest) and 100 units/ml penicillin and 0.1 mg/ml streptomycin (Life Technologies) at 37°C in a 5% CO_2_ humidified atmosphere.

### Transfection

pCMV-CREB133 vector was purchased from Takara Bio. The vector was transfected into MDCK cells using Attractene transfection reagent (Qiagen) in accordance with the manufacturer's protocol. After the transfected cells were grown in culture medium containing 10% FBS without antibiotics for 48 hours, the selective antibiotic G418 (Life Technologies) was added at 0.4 mg/ml. Culture medium containing G418 was changed every third day. Transfected clones were isolated and maintained using 0.4 mg/ml G418.

### Scratch Wound

MDCK cells were plated on glass-based 35 mm dishes (Iwaki), and were grown for 2–3 days before use. During the wounding experiments, the cells were maintained in 1.8 mM or 0.1 mM Ca^2+^ Ringer's solution containing 1% FBS. The Ca^2+^-free Ringer's solution contained 138 mM NaCl, 2.7 mM KCl, 1.06 mM MgCl_2_, 5.6 mM D-glucose, and 12.4 mM HEPES (pH 7.25). A stock solution of 100 mM CaCl_2_ was used to adjust the concentration of Ca^2+^.

The marker dyes, Alexa 488-dextran (10,000 MW) and fixable Alexa 488-dextran (10,000 MW) (Life Technologies), were dissolved at a concentration of 0.5 mg/ml in 1.8 mM Ca^2+^ Ringer's solution containing 1% FBS. MDCK cells were rinsed once with 1.8 mM Ca^2+^ Ringer's solution containing 1% FBS at 37°C before adding the above Alexa solution at 37°C along with various reagents. Then, the cells were wounded by slowly scratching multiple times with a sterile 27G needle in the presence of the marker dye [29]. The cells were allowed to stand for 5 minutes and then were returned to normal culture conditions. The marker dye enters only those cells that incur a cell membrane disruption, and is retained in wounded cells that successfully reseal.

### Membrane Resealing Assay

Cells were initially wounded by scratching in the presence of Alexa 488-dextran as described above. Twenty-four hours later, these cells were loaded with 1 µM calcein red-orange AM (Life Technologies) in the culture medium for 1 hour at 37°C. During the wounding experiments, the cells were maintained in 1.8 mM Ca^2+^ Ringer's solution containing 1% FBS, and changes in the fluorescence intensity of calcein red-orange were monitored with an LSM510 laser scanning confocal microscope (ver. 3.2; Carl Zeiss) equipped with Axiovert (C-Apochromat 40×/1.2 W Corr objective). To disrupt the plasma membrane, glass needles were made from Narishige G-1000 glass rods by pulling with a Narishige PC-10. Wounding of cells was performed using an InjectMan 5179 and a FemtoJet 5247 (Eppendorf) equipped with a microscope. The time setting for wounding was 0.3 seconds. A slowly declining fluorescence caused by photobleaching was subtracted from raw recordings before plotting the traces. A transient decrease of fluorescent intensity indicates successful resealing (see Figure 1A). A persistent decrease of fluorescent intensity as an indicator of dye loss from the cytosol indicates a resealing failure (see Figure 1B). To compare the timing of membrane resealing, reciprocal of resealing time was calculated, and termed “resealing rate” as described in previous studies [4], [8], [11], [13], [18], [19]. For cells that failed to reseal, the rate was defined as zero [18].

### Western Blotting

To stimulate phosphorylation of CREB, MDCK cells were treated with 100 µM forskolin, an activator of adenylyl cyclase, plus 1 µM IBMX (3-isobutyl-1-methylxanthine), an inhibitor of phosphodiesterase, for 15 minutes. Treated and non-treated cells were washed once with PBS, and lysed in M-PER Mammalian Protein Extraction Reagent (Thermo Scientific) containing 150 mM NaCl, Protease Inhibitor Cocktail (Sigma), and Halt Phosphatase Inhibitor Cocktail (Thermo Scientific) at 4°C. The lysates were centrifuged at 14,000 g for 5 minutes to pellet the cell debris. As controls, CREB Control Cell Extracts from SK-N-MC cells were obtained from Cell Signaling Technology. These samples were then separated by NuPAGE Novex 4–12% Bis-Tris gels (Life Technologies), and electrophoretically transferred to PVDF membrane. Primary antibody used in this study was anti-phospho-CREB (Ser133) (87G3) mAb (Cell Signaling Technology) that recognizes the peptide sequence containing phosphorylated Ser133 of CREB. This sequence occurs in the transcriptional activating domain of the CREB family of transcription factors such as ATF-1 [30], [31]. The antibody was diluted at 1∶1000, and incubated with the membrane at 4°C overnight with or without pre-incubation with phospho-CREB (Ser133) blocking peptide (Cell Signaling Technology). Immunoreactive bands were detected by WesternBreeze Immunodetection kit (Life Technologies).

### Immunofluorescent Staining

Cells, scratched in the presence of fixable Alexa 488-dextran as described above, were fixed with 4% formaldehyde in PBS 1 hour after the scratching. The cells were then permeabilized with 0.1% Triton X-100 in PBS for 5 minutes, and immunostained with anti-phospho-CREB antibody (1∶800 dilution) at 4°C overnight. Alexa 546 conjugated goat anti-rabbit IgG (Life Technologies) was used at 2 µg/ml for detection. Images were acquired with an LSM510 laser scanning confocal microscope. The fluorescent intensities of immunostaining were evaluated by ImageJ 1.46 g, a public domain image processing software from the National Institutes of Health.

### Statistic Analysis

Statistical comparisons were performed by Prism 5 (GraphPad Software). ANOVA with Bonferroni's multiple comparison test was used.
